# Expression of Aldehyde Dehydrogenase 1A1 in Relapse-Associated Cells in Acute Myeloid Leukemia

**DOI:** 10.3390/cells14131038

**Published:** 2025-07-07

**Authors:** Régis Costello, Garrett M. Dancik, Anaïs Dubiau, Lamia Madaci, Spiros Vlahopoulos

**Affiliations:** 1 APHM, Hematology and Cellular Therapy Department, Conception Hospital, 13005 Marseille, France; regis.costello@free.fr; 2 TAGC-Theories and Approaches of Genomic Complexity, INSERM, UMR1090, Parc Scientifique de Luminy, Aix Marseille University, 13009 Marseille, France; anais.dubiau.ad@gmail.com (A.D.); lamia.madaci@inserm.fr (L.M.); 3 Department of Computer Science, Eastern Connecticut State University, Willimantic, CT 06226, USA; dancikg@easternct.edu; 4 First Department of Pediatrics, National and Kapodistrian University of Athens, Thivon & Levadeias 8, Goudi, 11527 Athens, Greece

**Keywords:** acute myeloid leukemia, leukemia stem cells, stem-like cells, relapse, reactive oxygen species, aldehyde dehydrogenase, gene expression, nucleophosmin, enzyme inhibitor, clinical trial

## Abstract

In acute myeloid leukemia (AML) it is important to elucidate the biological events that lead from remission to relapse, which have a high probability of leading to an adverse disease outcome. The cancer stem cell marker aldehyde dehydrogenase 1 (ALDH1A1) is underexpressed in AML cells when compared to healthy cells, both at the RNA level and at the protein level, and at least in the former, both in the bone marrow and in peripheral blood. Nonetheless, ALDH1A1/ALDH1A2 activity increases in AML cells during disease relapse and is higher in adverse prognosis AML in comparison with favorable prognosis AML. Furthermore, especially in relapsed AML and in unfavorable AML, AML cells rich in ALDH1A1 can contain high levels of reactive oxygen species (ROS), in parallel with high ALDH1A1/2 activity. This metabolic feature is clearly incompatible with normal stem cells. The term “stem-like” therefore is useful to coin malignant cells with a variety of genetic makeups, metabolic programming and biomarkers that converge in the function of survival of clones sufficient to sustain, spread and re-establish neoplastic disease. Therefore, AML “stem-like” cells survive cancer treatment that eradicates other malignant cell clones. This fact differentiates AML “stem-like” cells from normal stem and progenitor cells that function in tissue regeneration as part of a distinct hierarchical order of cell phenotypes. The ODYSSEY clinical trial is a Phase I/II study designed to evaluate the safety, tolerability, pharmacokinetics, and pharmacodynamics of ABD-3001, a novel therapeutic agent, in patients with AML who have relapsed or are refractory to standard treatments. In this context, ABD-3001 is used as an inhibitor of cytosolic ALDH1 enzymes, such as ALDH1A1 and ALDH1A2.

## 1. Introduction

Cancer stem cells (CSCs) is a term that has been used to designate a distinct population of cells within tumors with capabilities of self-renewal and tumorigenicity. CSCs are considered pivotal in cancer progression, metastasis, relapse and tumor resistance to cytotoxic therapy, and they are often identified as a minority of the tumor cell population [1,2,3]. CSCs are now suggested as a potential source of relapse in any possible malignant tumor, with a distinct set of markers for stem cells from each main cancer type [1,2]. Acute myeloid leukemia (AML) stands out as one of the first cancer types for which the existence of tumor stem cells has been empirically validated [4,5].

The main characteristic of leukemia stem cell (LSC) populations is the ability to engraft and initiate leukemia in a recipient mouse (initiation), to grow out after re-transplantation into secondary recipients (self-renewal) and preferably in tertiary recipients [6,7]. This advance is due to the work of Lapidot et al., who in 1994 initially characterized LSCs in human AML as a small subset of leukemic cells capable of inducing leukemia when transplanted into immunodeficient mice [8]. In 1997, Bonnet and Dick determined that the LSC population was predominantly enriched in cells displaying the CD34+CD38- phenotype [2]. Yet leukemia-initiating capacity is detected also in CD34+CD38+ and in CD34- cell clones; which, however, in general, have a decreased capacity for leukemia initiation [4,6,7,9,10,11,12].

**Figure 1:**
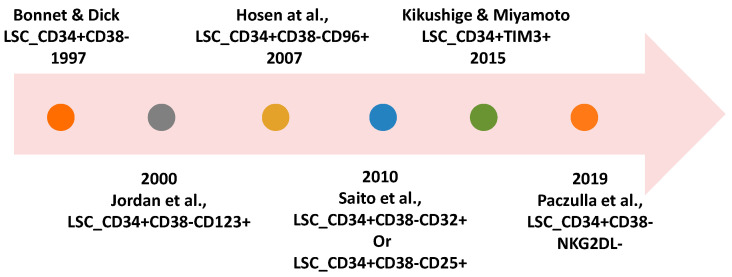
A few milestones in the study of stem-like cells for leukemia (Bonnet and Dick, 1997 [2]; Jordan et al., 2000, [22]; Hosen et al., 2007, [16]; Saito et al., 2010, [23]; Kikushige and Miyamoto, 2015, [15]; Paczulla et al., 2019, [21]).

Mirroring the hierarchical organization seen in normal hematopoiesis, leukemic clones are similarly structured, with self-renewing LSCs at the top, and more differentiated leukemic blasts further down the hierarchy, that lack the inherent ability to self-renew [13]. This CD34+CD38- subpopulation has become a specific marker of LSCs in AML, although some normal hematopoietic stem cells also share these markers. In addition, the presence of markers such as CD123 [14], TIM3 [15], CD96 [16], CD33 [17], CD244 [18], CLL1 [19] and CD47 [20], as well as the lack of NKG2DL [21], have been identified as distinguishing LSCs, paving the way for potential targeting (Figure 1).

These LSCs play a key role in resistance to chemotherapy and thus in triggering disease relapse [24,25]. Nevertheless, the fundamental mechanisms by which LSCs induce AML relapse have yet to be fully understood [26]. Efforts to target LSCs have emerged as a promising strategy for improving long-term survival rates in AML patients [27]. Consequently, the study of the distinctive biological features that are intrinsic to LSCs is of considerable importance in pharmaceutical research, for the formulation of precision therapies and for advancing our understanding of the complex molecular mechanisms underlying the disease [28,29]. However, the development of unique targeting strategies directed against these LSC-associated antigens is proving complex. Indeed, none of these antigens is exclusively expressed on AML cells, implying a risk of severe toxicity outside the targeted leukemic population. Moreover, clonal heterogeneity as well as antigen escape mechanisms could lead to the persistence of AML cells after treatment targeting a single antigen [30]. Fortunately, LSCs are distinguished by alterations in signaling pathways crucial for their survival and development, as well as for their ability to resist chemotherapy treatments. These signaling pathways include NFκB, PI3K/Akt/mTOR and JAK-STAT [31,32,33,34,35] together with protection mechanisms regarding oxidative stress [36] and autophagy [37,38].

In mouse xenograft models, it was shown that cells with metabolic properties that deviate from classical stem cells could resist cytarabine chemotherapy and essentially could reinitiate AML [39,40,41].

LSCs are also resistant to another classical AML drug, i.e., daunorubicine [42]. This resistance is related to an increased expression of multidrug-resistance genes (MRP/LRP). From these data, we can conclude that LSC are resistant to the two major drugs used for AML induction treatment in fit patients, i.e., the classical drug combination “3 + 7”.

It has been documented that cytarabine causes a partial differentiation of AML blast cells toward a monocytic phenotype [43]. Evidence suggests that a continuum of cellular phenotypes carries tumor-initiating cell properties, and that furthermore, malignant cells with differentiated phenotypes may generate CSC clones, such as the monocytic-like cells that are generated by cytarabine treatment of AML [1,10,44]. Characteristic mutations of the gene *NPM1* that are documented in AML are permissive to the activation of the oncogene *MYC*, to cell proliferation, and to generation of monocyte-like AML cells [45].

In particular, the cancer cells that are identified as “CSCs” based on distinct markers may be killed during cancer treatment, and therefore “CSCs” may appear to lose certain markers during the process of forced selection when subject to conditions that kill a substantial portion of the population of cancer cells that operate as “CSCs” [46]. But certain properties of the CSCs that are rooted in the genome and epigenome (modifications in chromatin), which determine the nature of the CSC function, appear to be enhanced during forced selection and corresponding cellular evolution in the host organism [47,48]. A likely reason for the emergence and prevalence of those CSC clones is that biomarker-positive CSCs are killed by cytotoxic agents and by the immune system. CSCs therefore do not exist as a distinct omnipotent type of AML cell, but arise by selective survival of a portion of AML cells resistant to killing by cytotoxic drugs and by the immune system. Before this forced selection, AML cells are not a hierarchically structured cell population but can be considered as a continuum of cell clones that differ from one another in terms of their chromatin modifications and phenotypes.

## 2. Biology Underlying AML Cell Dynamics

While a continuum in chromatin modifications that is accompanied by a corresponding continuum in phenotypes also manifests in the differentiation of normal hematopoietic cells [49,50,51], a distinguishing feature of malignant stem cells is the aberrant exposure of their genome, which disrupts biological function and enables them to respond differently to inflammatory stimuli and cell stress [52,53,54,55]. This aberrant exposure can be attributed to oncogene effects on the signaling apparatus that controls the epigenome [56,57]. Interestingly, however, early chromatin accessibility alterations within pre-leukemic hematopoietic stem cells cause defects in differentiation which correlate with adverse patient outcomes in AML [58]. Actually, chromatin profiles are prognostic of clinical response to proteasome inhibitor-containing chemotherapy in pediatric AML [59]. Thus, chromatin remodeling is an important factor in AML. This importance reflects a key difference between normal hematopoietic cells and AML cells.

Indeed, the physiological response to biological stress such as infections and cytotoxic drug-induced myeloablation cause molecular, cellular and metabolic changes in hematopoietic stem and progenitor cells at multiple levels of the hematopoietic hierarchy to drive accelerated production of the mature myeloid cells needed to resolve the initiating insult [60]. In contrast, malignant “stem-like” cells show a convergence of chromatin aberrations and corresponding changes in their transcriptome, even between cells with different genomes [1,61,62,63,64]. This does not mean that the cancer “stem-like” state is a fixed phenotype, but rather that it is a chromatin state that permits aberrant responses to cell stress, which may trigger the emergence of subclones with a high variation in phenotypes, biomarkers, and metabolic apparatus. The LSCs show the capacity to activate lineage-inappropriate signaling pathways to promote their growth. Moreover, AML patients harbor a population of quiescent LSCs which, upon cytokine stimulation, can emerge from quiescence to trigger relapse after therapy [65]. Cytokine expression helps LSCs to interact with mesenchymal cells, modulates and ultimately disrupts function of the human bone marrow niche to impair hematopoiesis and support diverse functions in LSCs [66,67].

Direct comparison of the normal bone marrow transcriptome with blastic bone marrow shows in the latter an upregulation of genes associated with inflammatory activation, including *NFKBIA, PTGS2, CD44, CXCL2, CXCL8, TGFβ1*, and *ANXA1*, and genes associated with extracellular matrix remodeling, including *LOLX2, TGFBI, COL5A3,* and *COL6A3.* On the other hand, genes encoding critical hematopoietic stem and progenitor cell (HSPC) regulatory proteins such as KITLG, CXCL12, IL7, the HSPC niche marker LEPR, and the preadipocyte marker LPL were significantly downregulated in the blastic bone marrow. In addition, a depletion of high-output hematopoietic stem cells was observed, while at least NPM1-mutant AML cells could maintain mTOR signaling, demonstrating that AML cells had, to a certain extent, increased resistance to cellular stress [68]. Nonetheless, the capacity to maintain active mTOR signaling is not uniform in AML-reinitiating cells.

In pediatric AML, the transmembrane receptor CD69 was found to be highly expressed in chemoresistant hematopoietic stem cell (HSC)-like populations, which were termed the “CD69+ HSC-like subpopulation”, manifesting suppression of the mTOR signaling pathway and promotion of cell quiescence and adhesion [69]. Another research team identified AML cells that were overexpressing CD69, had low levels of Ki67, high levels of NFκB, reduced proliferation, and enhanced colony-forming capacity [70]. In mice, there were two distinct populations of murine AML LSCs identified, with corresponding LSCs in human AML: those overexpressing the CD36 antigen that manifest increased proliferation, and those overexpressing CD69 that were capable of self-renewal and leukemia transplantation, but exhibiting decreased proliferation potential [71]. Therefore, in addition to the “monocyte-like”, partially differentiated AML cells that survive cytarabine chemotherapy and exhibit high CD36 expression [43], there exist conditions under which AML cells with suppressed CD36 expression and suppressed mTOR activity can reinitiate AML after treatment, such as the CD69-expressing quiescent HSC-like AML cells. It was later found that HSC-like AML cells have a proteome that resembles normal HSCs and are linked to a poor disease prognosis [72].

Thus, at least in AML, the possibility exists to encounter at least two different types of cells with a disease re-initiating potential after therapy.

Single-cell RNA sequencing shows a substantial variety in the developmental trajectories of AML cells [73]; however, convergence ensues after selective pressure. Single-cell RNA sequencing analysis demonstrated alterations in the metabolism of NPM1-mutant AML cells, i.e., low activity of oxidative phosphorylation in combination with high expression of the short non-coding RNA molecule *miR-126* associated with stemness and quiescence [74]. Those cells were enriched during chemotherapy and at relapse. In contrast, the MYC-positive NPM1-mutant cells have high activity of oxidative phosphorylation and a proliferative phenotype [74,75]. This is consistent with the extreme capacity of MYC protein to spur cell growth and proliferation by activating a comprehensive network of signaling and metabolic factors [76]. In AML, and possibly in other cancers, “stem-like” malignant cells may acquire a quiescent phenotype to survive cytotoxic stress and then revert to a variety of other phenotypes after changes in conditions [54]. At least in some cases, these quiescent cells may exhibit high expression of the enzyme aldehyde dehydrogenase ALDH1A1 [54,77]. The quiescent cancer cells may give rise to cells that possess identical genomes and possibly also epigenomes, but distinct phenotypes that respond differently to stress conditions: these cells may possess increased resistance to oxidative stress [55].

## 3. Expression of ALDH1A1 in AML

In a decisive demonstration of the power of interference with ALDH1-type enzymes, Venton et al. used the inhibitor DIMATE to demonstrate that inhibition of cytosolic retinaldehyde dehydrogenases interfered with viability of leukemia stem-like cells but not with normal stem cells [24]. It was subsequently proven that the inhibitor interfered with the capacity of leukemia cells to generate expanding clones resistant to oxidative stress [36].

Consistent with these results, Dancik et al. identified *ALDH1A1* as a gene that produces more RNA in samples from relapsed AML than primary AML [77], while the samples from favorable AML patients had lower *ALDH1A1* RNA expression [78]. Samples from patients with favorable AML also had a deficit in *ALDH1A1* RNA expression, according to a previous study by Gasparetto et al. [79].

These results regarding RNA expression have been further confirmed by Venton et al. [36], who analyzed aggregate ALDH1A1 and ALDH1A2 activity and reactive oxygen species (ROS) levels by flow cytometry. First, both ALDH1A1/2 activity and ROS levels correlated with the ELN22 prognostic groups (groups of prognostic classification based on molecular genetic features defined by European LeukemiaNet in year 2022), with higher ALDH1A1/2 and ROS levels in the unfavorable/intermediate groups, when compared to favorable AML. When the patients were classified on the basis of ALDH1A1/2 activity or ROS levels, higher values clearly distinguished patients with a shorter survival; the deficit in survival was especially notable for patients with samples that had higher ROS levels (OS 8 months for high levels vs. >24 for low levels in patient samples). Moreover and quite interestingly, both ALDH1A1/2 and ROS levels were higher in relapsed AML in comparison with AML at diagnosis. Although preliminary and in vitro results, these data could argue for an even higher efficacy of ALDH blockade in unfavorable or refractory/relapsed AML, while most leukemia treatments are usually less active in these subtypes, probably due to clonal selection of leukemia cells co-expressing chemotherapy resistance genes [80]. These data open an avenue for ALDH inhibition in AML treatment.

Importantly, patients with CD34+CD38- leukemia cells with high ALDH activity manifest a significantly lower complete remission rate, as well as poorer event-free and overall survival [81]. Interestingly, ROS are linked to activation of innate immunity [82]. Results from a single-cell study of pediatric AML samples suggested that activation of innate immune signaling in primitive cells (a) strongly associated with unfavorable prognosis and AML relapse and (b) promoted an advantage of clonal competition for AML cells, that enabled subsequent clonal expansion [83]. Thus, ALDH activity and ROS in primitive leukemia cells converge to a negative prognosis. However, this convergence identifies a critical vulnerability of AML “stem-like” cells.

**Figure 2:**
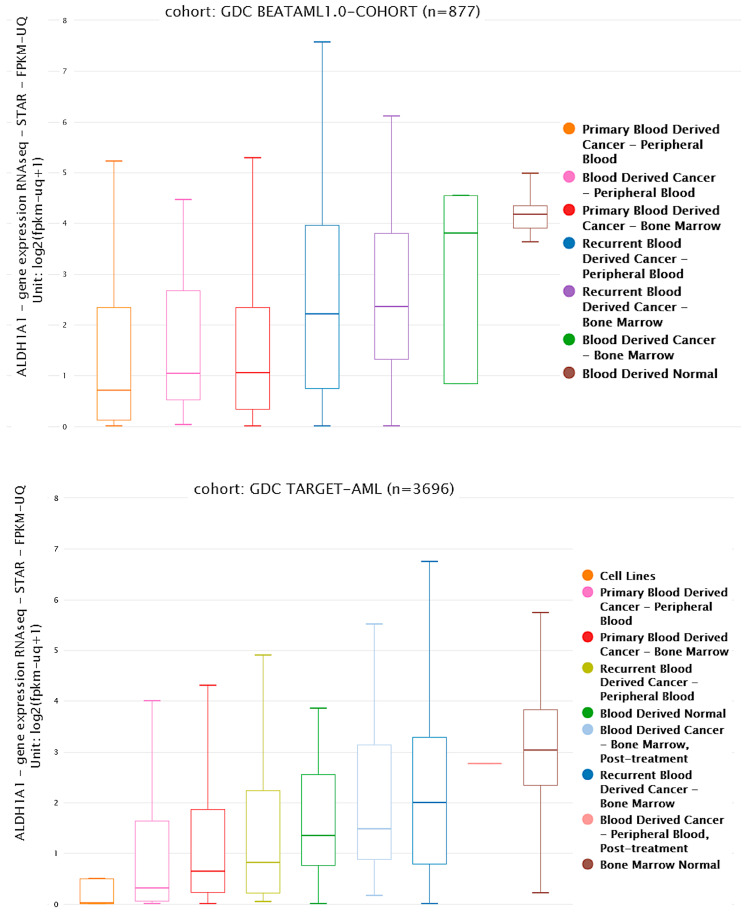
Expression of RNA from the gene *ALDH1A1* in patient samples from the BEAT AML and TARGET AML studies. Samples are divided by sample source and disease state. The f-values obtained from one way analysis of variance are 24.11, *p* = 0 (BEAT AML), and 96.06, *p* = 0 (TARGET AML).

Healthy stem cells do not have a deficiency in ALDH1A1 activity, and they express *ALDH1A1* in high levels at both the RNA and protein levels when compared to AML cells [12,72,79,84], which is evident in the analysis of data from the BEAT AML and TARGET studies using the UCSC Xena platform (https://xenabrowser.net/ accessed on 22 February 2025) (Figure 2) [85,86,87].

AML “stem-like” cells are therefore a less-than-matching equivalent to healthy hematopoietic stem cells, and it is only under certain conditions that are associated with a poor disease course that AML cells get enriched for ALDH1A1 expression. The lower expression of ALDH1A1 clearly identifies a crucial deficiency of AML cells and a potential vulnerability: DIMATE, in the pharmaceutical form of ABD-3001, is currently under study for relapsed and refractory AML in the clinical trial ‘ODYSSEY’ (NCT05601726). The ODYSSEY clinical trial concerns patients with AML who have relapsed or are refractory to standard treatments and cannot benefit from allogeneic stem cell transplantation. This clinical trial comprises two phases, with the first consisting of administration of a single dose. This first phase was used to determine the limiting doses. Compared with the levels effective in vitro for eliminating leukemic blasts while sparing healthy hematopoietic cells, the serum levels obtained at non-toxic doses appear to be entirely compatible with anti-leukemic efficacy (unpublished data). Although this was not the aim of a phase I trial, the therapeutically beneficial effects observed in one of the patients led to a new administration on a compassionate basis. At present, the second part of the trial is analyzing repeated administration of DIMATE once or twice a week for 12 weeks, with good hematological effects and general tolerability (preliminary unpublished data).

We previously postulated that in respect to metabolism and oxidative stress, there are at least two basic phenotypic states for leukemia stem-like cells; these extreme states present variable degrees of overlap in a given cell or in daughter clones [54]:

(1) a state of relative metabolic dormancy with varying degrees of cycling quiescence and a protection from oxidant stress mainly from a decreased generation of ROS, and secondarily from increased expression and activity of cytosolic retinaldehyde enzymes such as ALDH1A1 and ALDH1A2. It must be noted that quiescence can be induced in leukemia cells or in naive embryonic stem cells by inhibition of MYC, which, among several other effects, is accompanied by decreased one-carbon metabolism and decreased oxidant stress [88,89,90,91].
(2) a state of increased metabolic activity and high capacity for proliferation, characterized by high activity of transcription factors such as MYC, and protection from oxidant stress mainly through a number of potent antioxidant enzymatic systems. A MYC-dominated cellular phenotype should generate large amounts of formaldehyde and acetaldehyde through several metabolic processes [92,93,94,95,96]. Formaldehyde and acetaldehyde in mitochondria can be removed by ALDH2 [97,98]. In zebrafish cells, formaldehyde accumulation was shown to cause organelle damage [99].

The turnover of MYC is controlled also by nucleophosmin, the protein encoded by gene *NPM1*, which is frequently mutated in AML, extending MYC activity and effectively enhancing proliferation [57,100,101]. One method to interfere with the effects of *NPM1* mutation is via intervention targeting the scaffold protein menin [102]. It was shown that mutated NPM1 protein, remaining in the cytoplasm, enhances leukemia progression via permitting induction of *HOX* gene expression [103]. In fact, nuclear relocalization or degradation of mutant NPM1 induces cell growth arrest and differentiation in primary AML cells. It was shown that NPM1-mutant protein conferred on AML cells a growth advantage [103]. Reinforcing the notion of metabolic stress, in patients with NPM1 mutations, vitamin C and D supplementation was significantly and independently associated with better overall survival (hazard ratio [HR], 0.52; 95% confidence interval [CI], 0.30–0.90; *p*= 0.019), compared with patients with wild-type NPM1 (HR, 1.01; 95% CI, 0.68–1.51; *p* = 0.95) [104]. In mice, vitamin C was shown to abrogate bone marrow stroma protection of AML cells from ROS without affecting the viability of normal stem cells [105].

Published microarray-based studies of AML patient samples (Gene Expression Omnibus datasets GSE1159, GSE12417, GSE37642, GSE6891, GSE8970 analyzed via the platform KM Plotter [106] https://kmplot.com/analysis/index.php?p=background, accessed on 30 October 2024), find that stratification of patients based on *ALDH2* RNA expression yields a higher hazard ratio than stratification based on *ALDH1A1* RNA expression, for both overall, event-free, and post-progression free survival in NPM1-mutant AML (Table 1). Here, a high HR value (HR > 1) indicates that high expression is associated with poor prognosis. Furthermore, the hazard ratio for *ALDH2* RNA expression is higher in NPM1-mutant patients compared to wild-type, which is not the case with *ALDH1A1*.

What makes this result even more compelling is the fact that when we look individually at the datasets with known NPM1 mutants and stratify patients based on *ALDH1A1* or *ALDH2* expression, we note that the difference between NPM1 wild-type and mutant AML generally holds true. In both datasets, stratification of patients with NPM1 mutations based on *ALDH2* RNA expression yields a higher hazard ratio than stratification based on *ALDH1A1* RNA expression, for both overall and event-free survival (Table 2). For convenience, we provide absolute numerical values for those published datasets as Supplementary Materials.

The most common clinically approved inhibitor precursor for ALDH2, disulfiram (used for the maintenance of abstinence from alcohol because it is metabolized into potent ALDH2 inhibitors in the organism), causes accumulation of toxic formaldehyde in cells that operate the mitochondrial respiratory chain with high activity and also causes accumulation of acetaldehyde in cells [107,108,109,110,111]. This would make disulfiram an important translational agent for AML cells resistant to ALDH1 (retinaldehyde dehydrogenase) inhibitors because ALDH1 inhibitors would tend to have a stronger effect against more quiescent leukemia cells. This could have a broader significance due to a number of alterations that bestow cancer cells with the capacity to respond to cytokines or to favorable changes in conditions with a rapid increase in metabolic capacity and macromolecule turnover and repair [55,112,113,114].

Malignant “stem-like” cells, therefore, have substantial deviations from normal stem cell features. However, the malignant “stem-like” cells have a substantial capacity to give rise to diverse phenotypes of cells with tumor-initiating potential, due to aberrations in the patterns of chromatin accessibility, which controls the capacity of transcription-activating proteins to induce the expression of genes with oncogenic potential [55,115].

Recently, a review was published that summarized single-cell studies conducted on leukemia, with focus on four key aspects: (1) leukemia’s clonal architecture, (2) frameworks to determine leukemia subtypes, (3) tumor microenvironment and (4) the drug-resistant mechanisms of leukemia [116]. This study reinforced the notion that there is not a single type of leukemia stem cell that initiates relapse; rather, there appears to be a convergence of properties that lead to relapse, which is to a certain extent independent from the biomarkers of the cells that are involved in the relapse; this challenges the notion of a fixed, omnipotent “leukemia stem cell” [40,117].

Therefore, in contrast to a hypothetical, hierarchical model of gradual development of malignant cell clones, the malignant cells that function as “stem-like” cells are essentially malignant cell clones that survive certain conditions that kill other leukemia cells. This survival depends on the proteins that leukemia “stem-like” cells express. To make the distinction between leukemia “stem-like” cells and normal stem cells even more obvious, the clones that can function as leukemia “stem-like” cells change under conditions of changes in ROS generation which allow cells with differences in ALDH enzyme expression to survive while other leukemia cell clones are eliminated.

**Figure 3:**
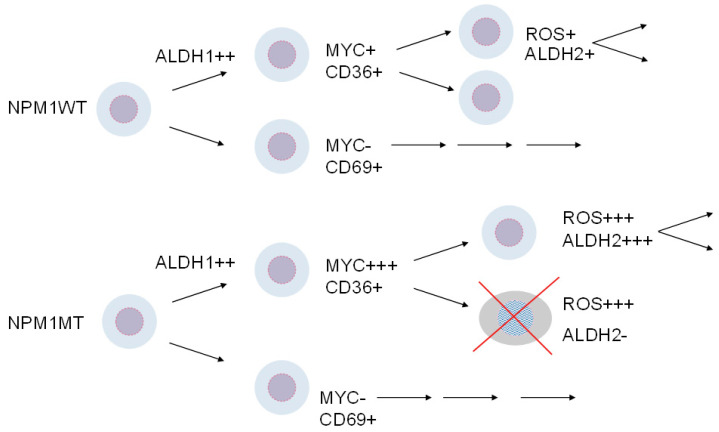
A schematic depiction of a working model for AML relapse. Expression and activity of ALDH1, which encompasses for example cytosolic enzymes ALDH1A1 and ALDH1A2, is a limiting factor that is needed for AML cell transitions from low ROS to high ROS. In contrast, for a longer-term survival under conditions of high ROS activity, the mitochondrial enzyme ALDH2 protects AML cells when they generate an excess of formaldehyde and acetaldehyde. The markers CD36 and CD69 are used here solely to designate a proposed indicative course of cellular evolution.

A working model for AML relapse recognizes the key role of ALDH1A1 during transitions of leukemia cells from ROS-low to ROS-high conditions, in contrast to the mitochondrial enzyme ALDH2, which is crucial under conditions of lasting generation of ROS. NPM1 mutation enables a higher MYC activity and higher levels of inflammatory cytokines and ROS. These conditions are detrimental for the function of the bone marrow. AML cells with sufficiently high ALDH2 activity survive formaldehyde and acetaldehyde generation and can cause more damage to the bone marrow (Figure 3).

One interesting pathway for long-term survival of quiescent leukemia-initiating cells is the suppression of mitochondrial complex I activity, with a concomitant increase in cell dependency on fatty acid oxidation. This was observed in CD34-negative chronic myelogenous leukemia cells that were resistant to the tyrosine kinase inhibitor imatinib [118]. The specific CREB-binding protein (CBP)/β-catenin antagonist ICG-001 initiated the differentiation of LSC, decreased chromatin accessibility and, by increasing expression of mitochondrial complex I, CD34, CD38 and BCR-ABL1, was able to resensitize leukemic cells to imatinib.

Despite the presence of vast transcriptional heterogeneity at the single-cell level, pathway analysis identified common signaling networks involving metabolism, apoptosis and chemokine signaling that evolved during AML progression and became a signature of relapse samples [119]. The hexosamine biosynthetic pathway, which regulates the O-GlcNAcylation of cytoplasmic and nuclear proteins, was linked to NFκB activity in AML cells at the single-cell level and was tied to the survival of those malignant cells [120]. Inhibiting NFκB in leukemia cells with Bruton tyrosine kinase (BTK) inhibitor ibrutinib had a potent impact, allowing only a quiescent population of malignant cells to survive treatment [121]. An early reappearance of ibrutinib resistant clones can be linked to expression of the MYC protein or of expression of the innate immunity activator TLR9 [122]. The *MYC* gene is regulated also at the level of chromatin accessibility [123].

All this information reinforces the notion that a flexibility exists in the types of cells that may function as the root of relapsed-refractory malignant disease, with the important inter-conversion between quiescent and proliferative phenotypes. The ease of inter-conversion, based on the studies conducted to date, suggests that chromatin accessibility enables rapid changes in gene expression, which cause dramatic alterations in the cells’ metabolic and proliferative potential, whereby chromatin accessibility tended to be more open when comparing malignant lineages with their normal counterparts [52,54,124]. Chromatin accessibility revealed a continuum of stem, progenitor, and lineage-restricted cell priming [52]. This is important, because the acquisition of a rich differentiation spectrum in the population of AML cells not only contributes to the generation and maintenance of leukemic stem cells, but the capacity to generate diverse phenotypes may also bring distinct barriers to therapeutic responses, which can lead to persistence of AML [52].

In essence, in contrast to normal stem cells, AML LSCs manifest after positive selection of malignant clones that are “fit” for stem-like function under certain conditions, and not as permanent origins of a natural hierarchy.

Therefore, the term “stem cell”, when referring to AML cells, is associated with stem cell function in terms of a quasi-opportunistic source of daughter AML clones and a limited potential of generation of differentiated cells. However, AML LSCs do not function as fully matching equivalents of normal healthy stem cells in terms of cellular biochemistry, gene expression, and interactions with their microenvironment [80].

## 4. Conclusions

Research has shown that there exist conditions when malignancy can recur through cells that are largely devoid of the main stem cell biomarkers. It has been proposed that gradual exposure to stress can improve a cell population’s fitness by allowing cells to “learn” and fine-tune stress regulatory networks, priming stable adaptive states while acquiring new metabolic dependencies [125]. Cellular adaptation is proposed to occur through “a step-wise assembly of gene expression programs and epigenetically reinforced cell states underpinned by phenotypic plasticity, adaptation to stress and metabolic reprogramming”, transitions that manifest as a “resistance continuum”. However, already early in the resistance continuum, drug-tolerant persistent cells may depend on stress-related programs and chromatin remodeling [125]. Thus, different epigenetic programs may enable acquisition of properties that allow cancer cell adaptation. Therefore, a variety of phenotypes of cells have the capacity to re-initiate and to propagate leukemia, as de facto “stem-like” cells, and these cells may possess stemness markers or may be negative for established stem cell markers [6,126].

Adding to this fact is the observation that the cancer stem cell marker ALDH1A1 is underexpressed in AML cells when compared to healthy cells [72], both at the RNA level and at the protein level, and at least in regard to the RNA level, both in the bone marrow and in peripheral blood. At least at the RNA level, *ALDH1A1* abundance increases in AML cells during disease relapse (recurrence). Furthermore, AML cells rich in ALDH1A1 can contain high levels of ROS, which is a metabolic feature clearly incompatible with normal stem cells [36]. The term “stem-like”, therefore, is useful to describe malignant cells in AML, with a variety of genetic makeups, metabolic programming, and biomarkers that converge in the function of survival of clones sufficient to sustain, spread and re-establish neoplastic disease.

The fact that these “stem-like” cells are positively selected after cancer treatment eradicates other malignant cell clones makes them different from normal stem and progenitor cells that are known to operate within a fixed hierarchy and defined function.

## Figures and Tables

**Table 1:** Stratification of AML patients based on *ALDH1A1* and *ALDH2* RNA expression.

NPM1MT (HR, P, FDR)	NMP1 WT (HR, P, FDR)	AML TOTAL (HR, P, FDR)	OVERALL SURVIVAL
1.21, 0.3, 100%	1.99, 6.6 × 10^−10^, 1%	1.47, 1.3 × 10^−10^, 1%	*ALDH1A1*
2.19, 0.00012, 1%	2.08, 4.4 × 10^−9^, 1%	1.81, <1 × 10^−16^, 1%	*ALDH2*
NPM1MT (HR, P, FDR)	NMP1 WT (HR, P, FDR)	AML TOTAL (HR, P, FDR)	EVENT-FREE SURVIVAL
0.79, 0.26, 100%	2.43, 1.8 × 10^−9^, 1%	1.7, 2.1 × 10^−6^, 1%	*ALDH1A1*
2.35, 0.00029, 2%	1.98, 1.1 × 10^−5^, 1%	1.75, 6.1 × 10^−5^, 1%	*ALDH2*
NPM1MT (HR, P, FDR)	NMP1 WT (HR, P, FDR)	AML TOTAL (HR, P, FDR)	POST-PROGRESSION SURVIVAL
1.53, 0.097, 100%	1.54, 0.0083, >50%	1.61, 0.00063, 5%	*ALDH1A1*
1.95, 0.0031, 10%	1.54, 0.016, >50%	1.43, 0.0056, 50%	*ALDH2*

HR, hazard ratio; FDR, false discovery rate.

**Table 2:** Stratification of patients from the studies GSE1159 and GSE6891, based on *ALDH1A1* and *ALDH2* RNA expression.

NPM1MT (HR, P, FDR)	NMP1 WT (HR, P, FDR)	AML TOTAL (HR, P, FDR)	OVERALL SURVIVAL
			GSE1159
1.25, 0.46, 100%	2.37, 3.7 × 10^−6^, 1%	1.62, 0.0014, 20%	*ALDH1A1*
1.94, 0.024, >50%	2.31, 8.3 × 10^−6^, 1%	2.08, 4.4 × 10^−9^, 1%	*ALDH2*
			GSE6891
1.27, 0.28, 100%	1.92, 2.3 × 10^−6^, 1%	1.51, 0.00013, 1%	*ALDH1A1*
2.62, 0.00069, 5%	2.06, 7 × 10^−6^, 1%	1.92, 4.3 × 10^−8^, 1%	*ALDH2*
NPM1MT (HR, P, FDR)	NMP1 WT (HR, P, FDR)	AML TOTAL (HR, P, FDR)	EVENT-FREE SURVIVAL
			GSE1159
0.63, 0.18, 100%	2.4, 5 × 10^−4^, 3%	1.6, 0.021, >50%	*ALDH1A1*
3.19, 0.022, 50%	2.49, 0.00039, 2%	1.9, 0.0021, 20%	*ALDH2*
			GSE6891
0.71, 0.2, 100%	2.47, 5.9 × 10^−7^, 1%	1.82, 9.9 × 10^−6^, 1%	*ALDH1A1*
2.84, 0.0016, 10%	1.84, 0.0014, 10%	1.6, 0.00098, 10%	*ALDH2*
NPM1MT (HR, P, FDR)	NMP1 WT (HR, P, FDR)	AML TOTAL (HR, P, FDR)	POST-PROGRESSION SURVIVAL
			GSE1159
2.05, 0.14, 100%	1.79, 0.04, >50%	1.53, 0.05, >50%	*ALDH1A1*
1.82, 0.22, 100%	2.12, 0.0087, 20%	1.49, 0.09, 100%	*ALDH2*
			GSE6891
1.48, 0.2, 100%	1.71, 0.0079, 50%	1.7, 0.0012, 10%	*ALDH1A1*
2.23, 0.0048, 10%	1.69, 0.029, >50%	1.5, 0.014, >50%	*ALDH2*

## Data Availability

Data from the published studies BEAT AML [85] and TARGET [86] were accessed using the platform UCSC Xena [87] (https://xenabrowser.net/, accessed on 22 February 2025). Data deposited in Gene Expression Omnibus (public functional genomics data repository supporting MIAME-compliant data submissions at https://www.ncbi.nlm.nih.gov/geo/, accessed on 30 October 2024) from the published studies GSE1159 [127], GSE12417 [128], GSE37642 [129], GSE6891 [130,131], GSE8970 [132] were reviewed with the help of the online analysis platform KM Plotter [106] (https://kmplot.com/analysis/index.php?p=background; accessed on 30 October 2024).

## Supplementary Materials

The following supporting information can be downloaded at: https://www.mdpi.com/article/10.3390/cells14131038/s1, Compressed folders OS, EFS, POST_PROGRESSION_SURVIVAL.

## Abbreviations

The following abbreviations are used in this manuscript:

BEAT AML	master clinical trial “Beat AML”
ELN […]	European LeukemiaNet [year]
DIMATE	Dimethyl AmpalThiolester
KM	Kaplan Meier
NCT […]	National Clinical Trials [number]
O-GlcNAcylation	O-linked β-N-acetylglucosamine modification
OS	Overall Survival
TARGET	Therapeutically Applicable Research to Generate Effective Treatments
TLR9	Toll-like receptor 9
