# Supplementary figures and images for: Expression of Aldehyde Dehydrogenase 1A1 in Relapse-Associated Cells in Acute Myeloid Leukemia
Source: Cells. 2025 Jul 7;14(13):1038. doi: 10.3390/cells14131038 (PMC12249481; doi:10.3390/cells14131038)

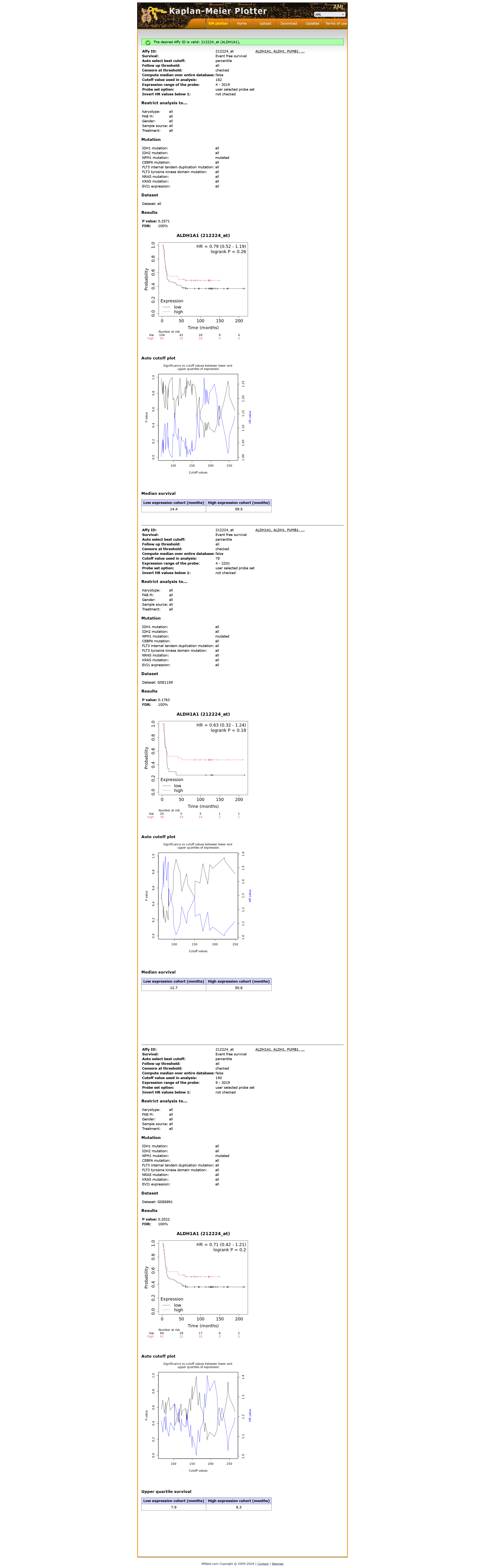

Supplement: Supplementary file 1 [file cells-14-01038-s001.zip › ABSOLUTE_VALUES_KM/EFS/ALDH1A1_EFS_NPM1MUT.png]

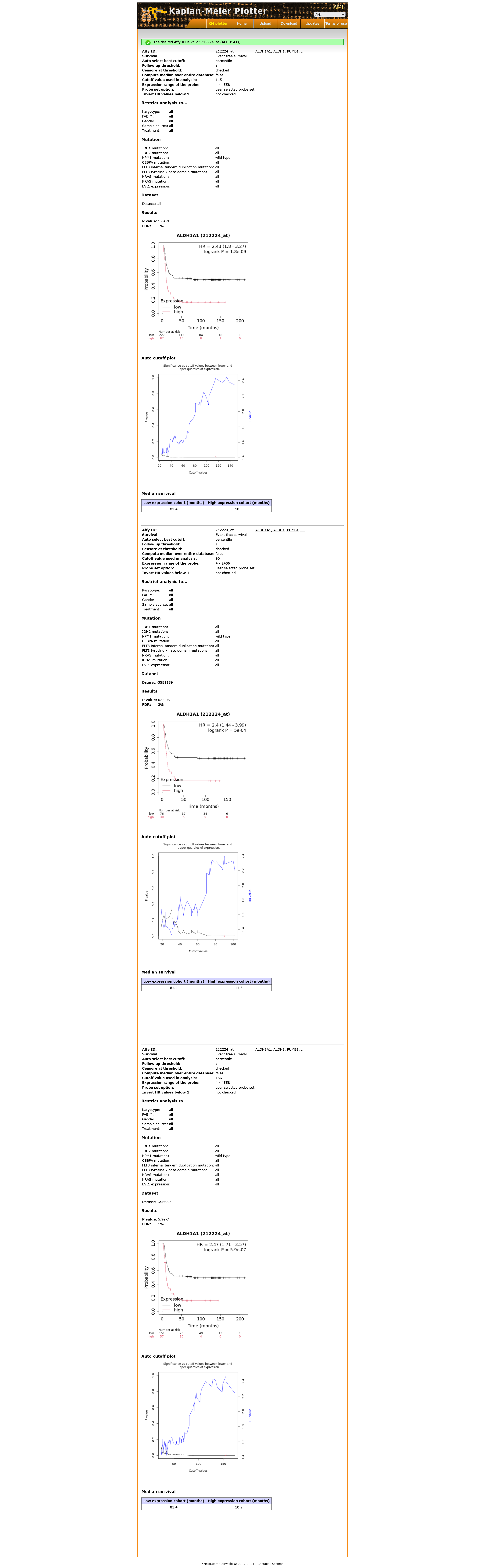

Supplement: Supplementary file 1 [file cells-14-01038-s001.zip › ABSOLUTE_VALUES_KM/EFS/ALDH1A1_EFS_NPM1WT.png]

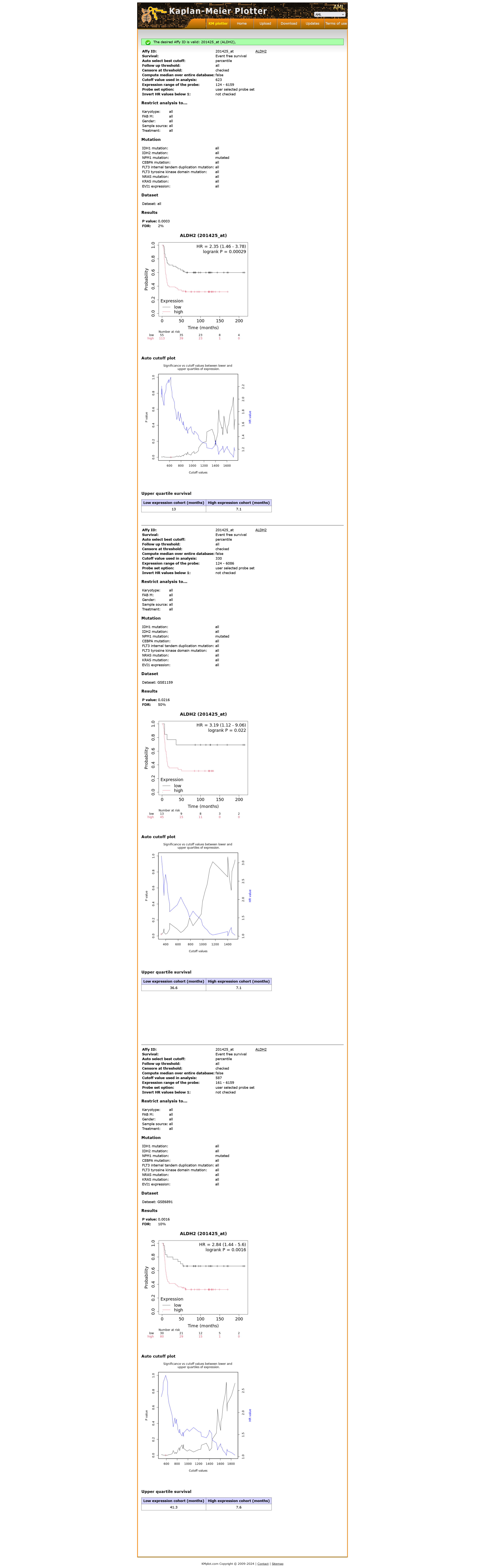

Supplement: Supplementary file 1 [file cells-14-01038-s001.zip › ABSOLUTE_VALUES_KM/EFS/ALDH2_EFS_NPM1MUT.png]

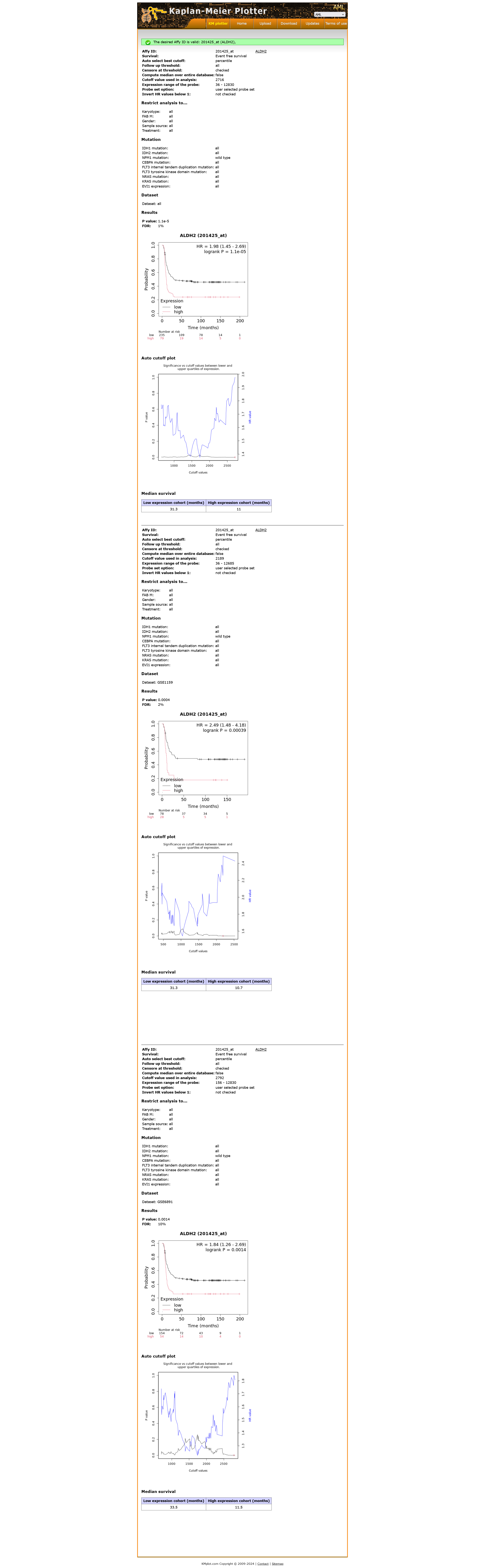

Supplement: Supplementary file 1 [file cells-14-01038-s001.zip › ABSOLUTE_VALUES_KM/EFS/ALDH2_EFS_NPM1WT.png]

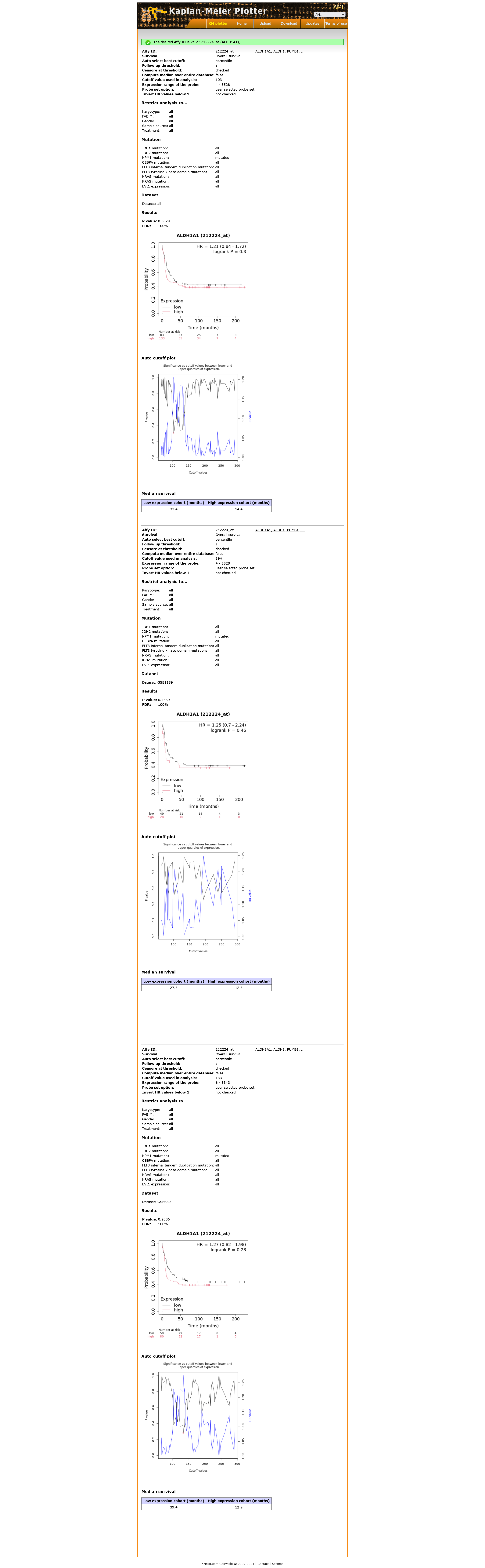

Supplement: Supplementary file 1 [file cells-14-01038-s001.zip › ABSOLUTE_VALUES_KM/OS/ALDH1A1_OS_NPM1MUT.png]

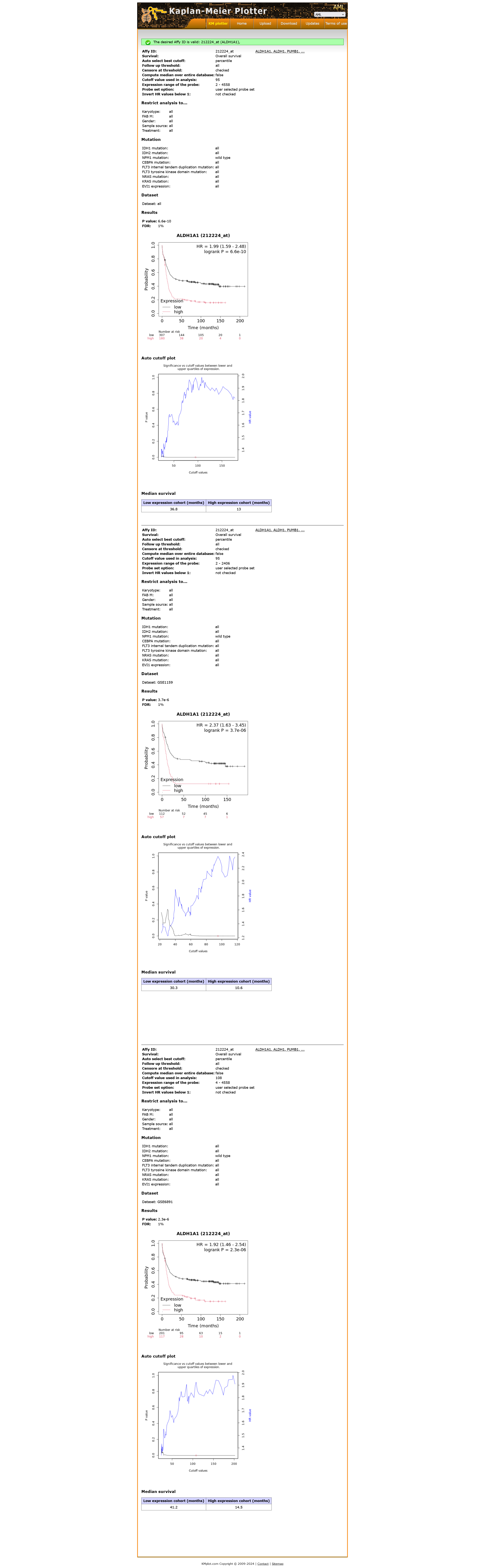

Supplement: Supplementary file 1 [file cells-14-01038-s001.zip › ABSOLUTE_VALUES_KM/OS/ALDH1A1_OS_NPM1WT.png]

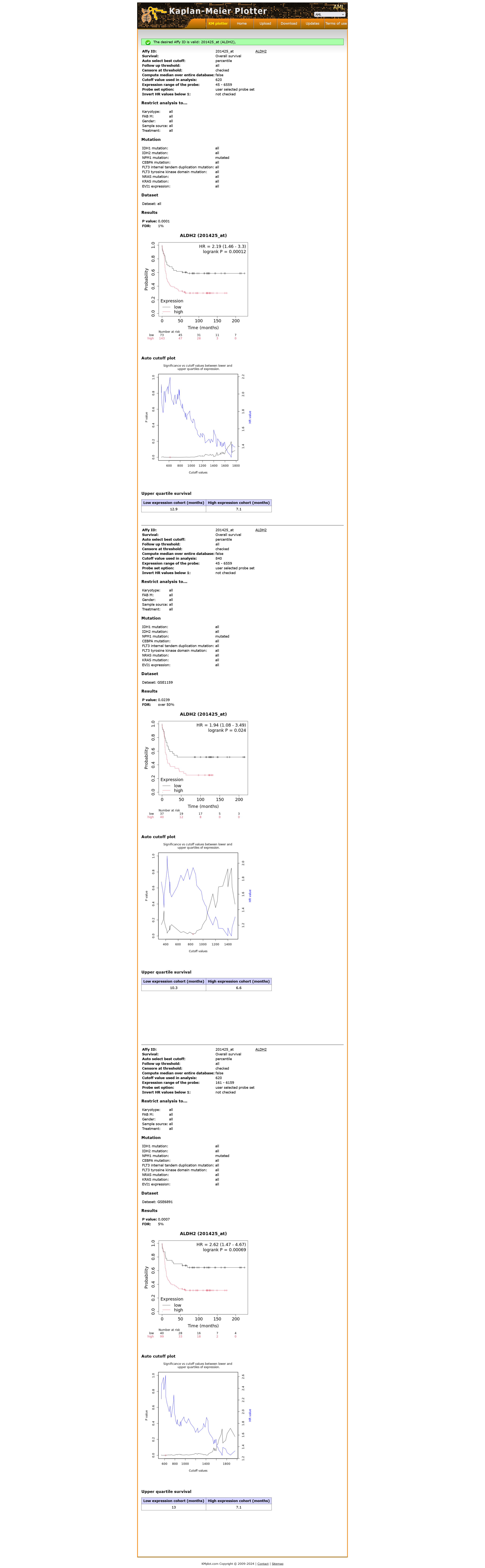

Supplement: Supplementary file 1 [file cells-14-01038-s001.zip › ABSOLUTE_VALUES_KM/OS/ALDH2_OS_NPM1MUT.png]

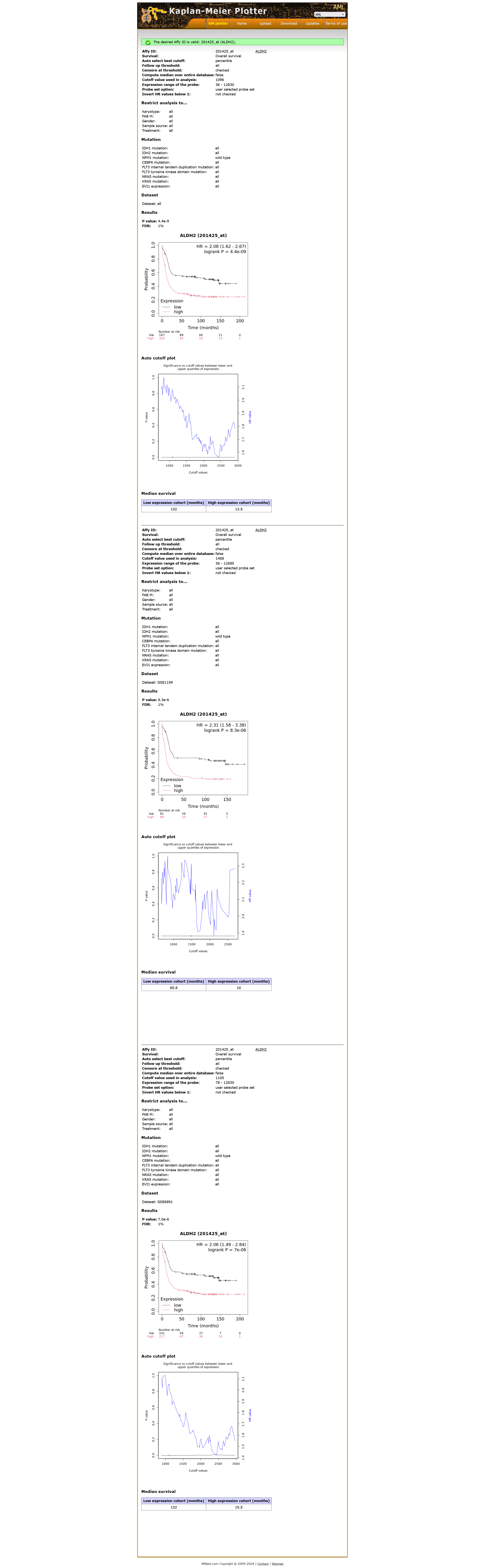

Supplement: Supplementary file 1 [file cells-14-01038-s001.zip › ABSOLUTE_VALUES_KM/OS/ALDH2_OS_NPM1WT.png]

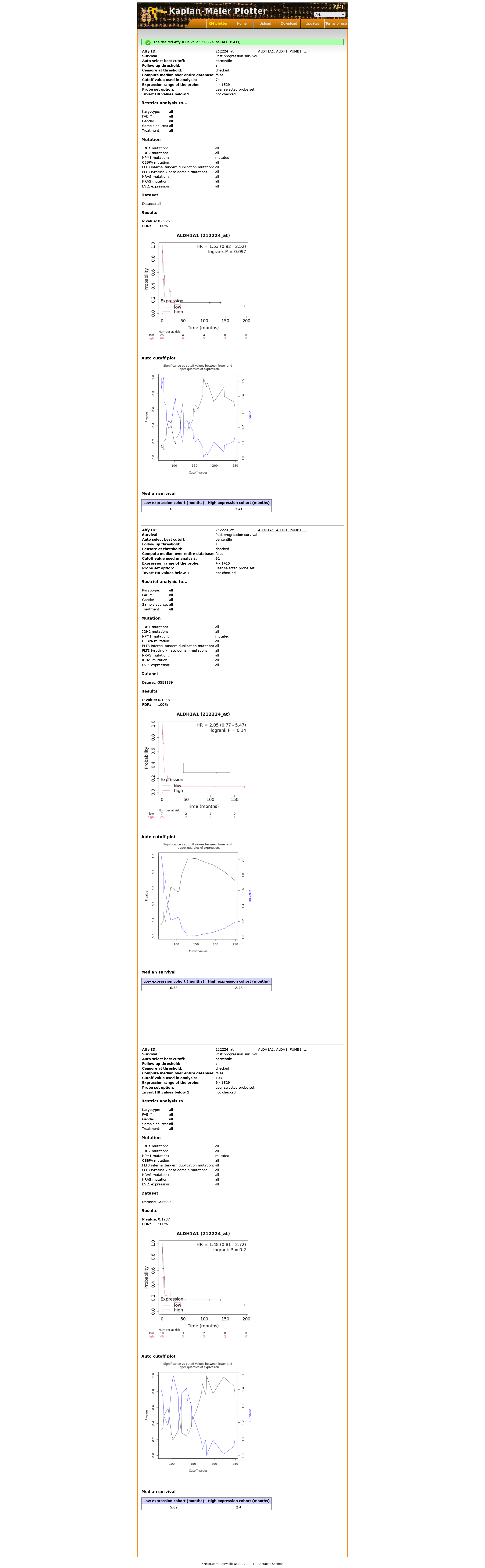

Supplement: Supplementary file 1 [file cells-14-01038-s001.zip › ABSOLUTE_VALUES_KM/POST_PROGRESSION_SURVIVAL/ALDH1A1_POSTPROGRESSIONSURVIVAL_NPM1MUT.png]

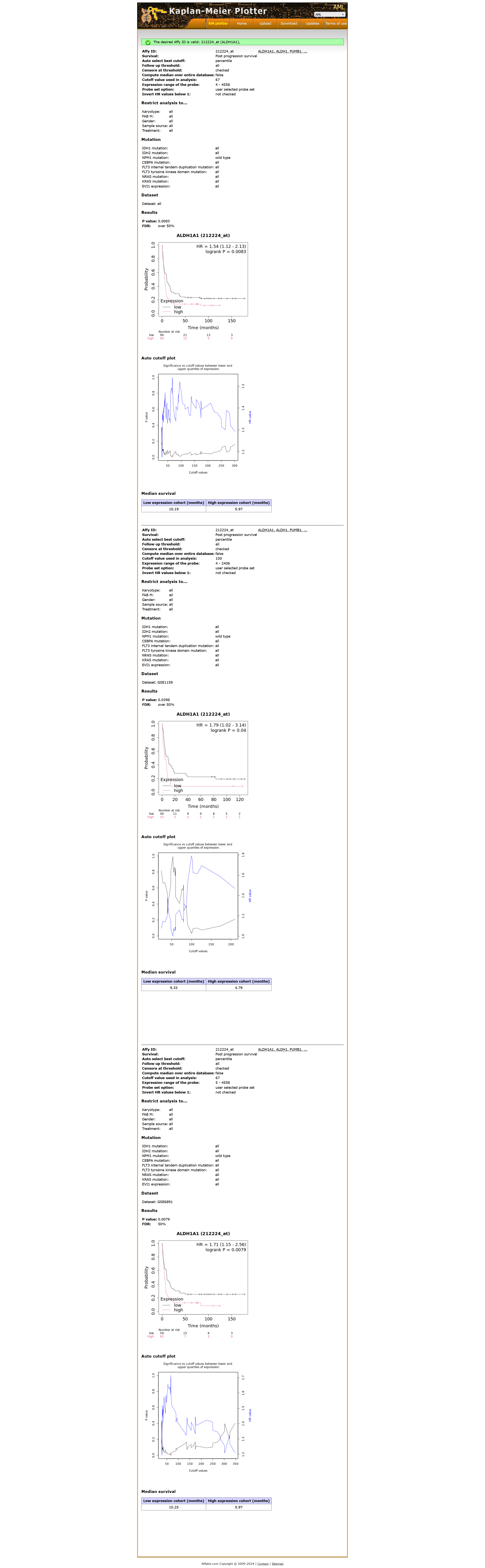

Supplement: Supplementary file 1 [file cells-14-01038-s001.zip › ABSOLUTE_VALUES_KM/POST_PROGRESSION_SURVIVAL/ALDH1A1_POSTPROGRESSIONSURVIVAL_NPM1WT.png]

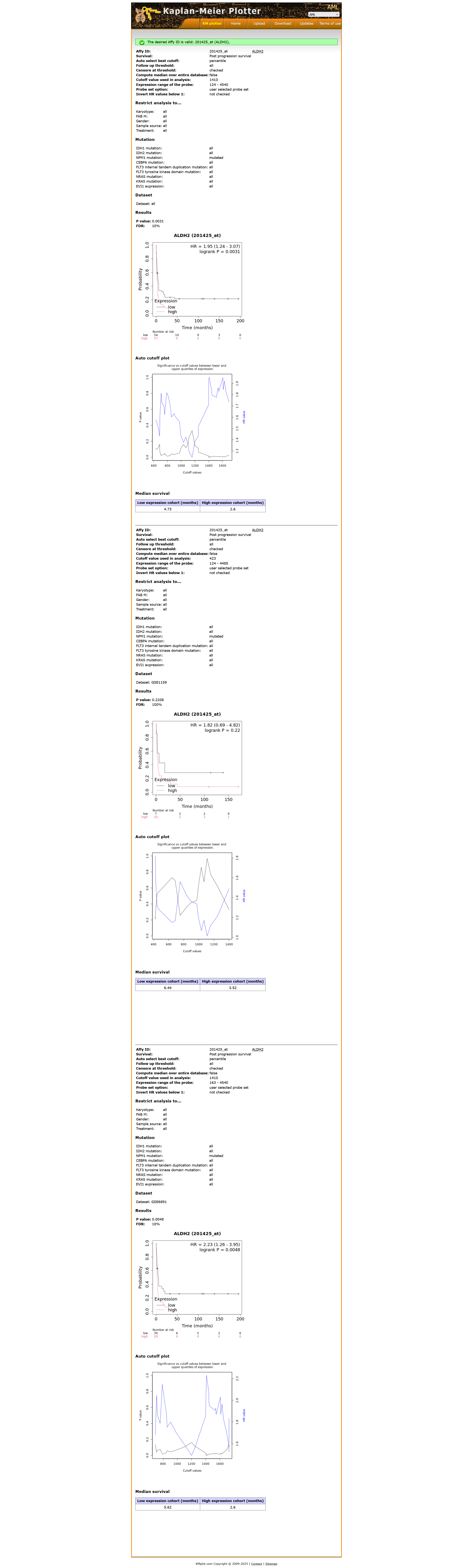

Supplement: Supplementary file 1 [file cells-14-01038-s001.zip › ABSOLUTE_VALUES_KM/POST_PROGRESSION_SURVIVAL/ALDH2_POSTPROGRESSIONSURVIVAL_NPM1MUT.png]

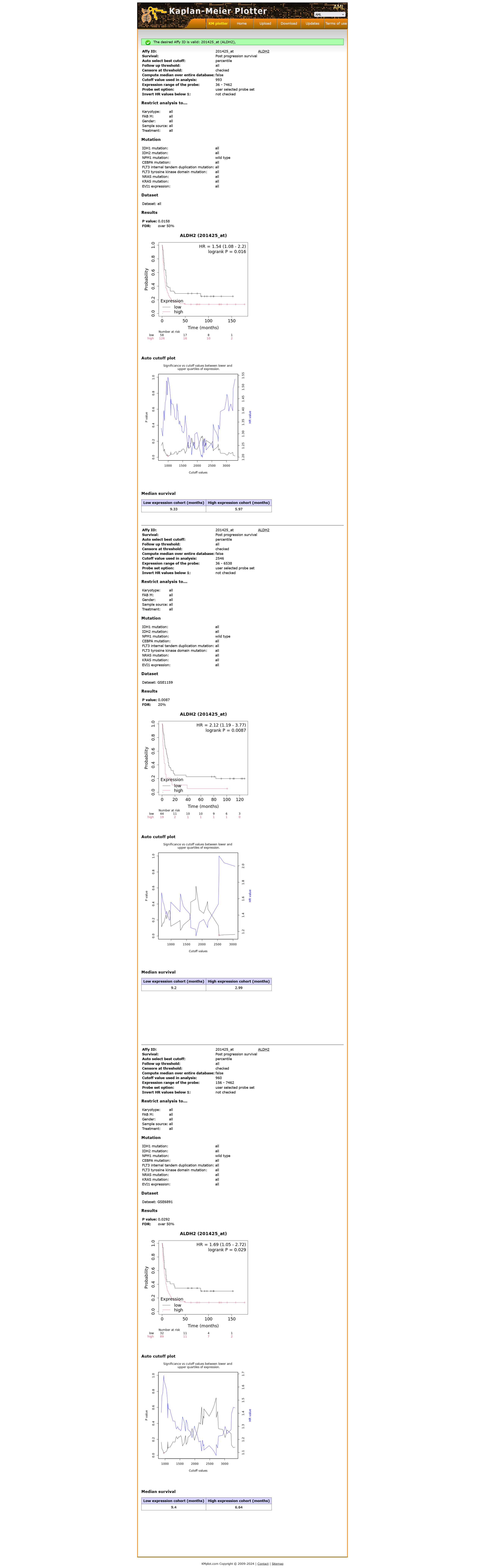

Supplement: Supplementary file 1 [file cells-14-01038-s001.zip › ABSOLUTE_VALUES_KM/POST_PROGRESSION_SURVIVAL/ALDH2_PostProgressionSurvival_NPM1wt.png]
